# The application of multi-criteria decision analysis in evaluating the value of drug-oriented intervention: a literature review

**DOI:** 10.3389/fphar.2024.1245825

**Published:** 2024-04-24

**Authors:** Pengli Su, Kai Zhi, Huanhuan Xu, Jing Xiao, Jun Liu, Zhong Wang, Qiong Liu, Yanan Yu, Haixia Dang

**Affiliations:** ^1^ Institute of Basic Research in Clinical Medicine, China Academy of Chinese Medical Sciences, Beijing, China; ^2^ China Academy of Chinese Medical Sciences, Beijing, China; ^3^ School of Public Health, Nantong University, Nantong, Jiangsu, China

**Keywords:** multi-criteria decision analysis, drug value, criteria, weighting techniques, scoring techniques

## Abstract

**Objectives:** Multi-Criteria Decision Analysis (MCDA) has gained increasing attention in supporting drug risk-benefit assessment, pricing and reimbursement, as well as optimization of clinical interventions. The objective of this study was to systematically collect and categorize evaluation criteria and techniques of weighting and scoring of MCDA for drug value assessment.

**Methods:** A systematic review of the literature was conducted across seven databases to identify articles utilizing the MCDA frameworks for the evaluation of drug value. Evaluation criteria mentioned in the included studies were extracted and assigned to 5 dimensions including clinical, economic, innovative, societal and humanistic value. A descriptive statistical analysis was performed on the identified drug value evaluation criteria, as well as the weighting and scoring techniques employed. The more a criterion or technique were mentioned in articles, the more important we consider it.

**Results:** Out of the 82 articles included, 111 unique criteria were identified to evaluate the value of drug. Among the 56 unique criteria (448 times) used to measure clinical value, the most frequently mentioned were “comparative safety/tolerability” (58 times), “comparative effectiveness/efficacy” (56 times), “comparative patient-perceived health/patient reported outcomes” (37 times), “disease severity” (34 times), and “unmet needs” (25 times). Regarding economic value measurement, out of the 20 unique criteria (124 times), the most frequently utilized criteria were “cost of intervention” (17 times), “comparative other medical costs” (16 times), and “comparative non-medical costs” (18 times). Out of the 10 criteria (18 times) for assessing innovative value, “a novel pharmacological mechanism” was the most frequently mentioned criterion (5 times). Among the 22 criteria (73 times) used to measure societal value, “system capacity and appropriate use of intervention” was the most frequently cited criterion (14 times). Out of the 3 criteria (15 times) utilized to measure humanistic value, “political/historical/cultural context” was the most frequently mentioned criterion (9 times). Furthermore, 11 scoring and 11 weighting techniques were found from various MCDA frameworks. “Swing weighting” and “a direct rating scale” were the most frequently used techniques in included articles.

**Conclusion:** This study comprehensively presented the current evaluation dimensions, criteria, and techniques for scoring and weighting in drug-oriented MCDA articles. By highlighting the frequently cited evaluation criteria and techniques for scoring and weighting, this analysis will provide a foundation to reasonably select appropriate evaluation criteria and technique in constructing the MCDA framework that aligns with research objectives.

## 1 Background

In recent decades, several “value frameworks” have been applied to evaluate the drug value in the health technology assessment (HTA) in the world (de Andrés-Nogales et al., 2020; de Andrés-Nogales et al., 2021; Hummel et al., 2012). These frameworks are employed to facilitate dynamic drug supervision, promote clinical rational drug use, adjust coverage and reimbursement decisions, and support drug marketing (Yu et al., 2023). In China, HTA assessment evidence based on comprehensive drug evaluations has also been used to update the national reimbursement drug list since 2017 (Chen et al., 2023). Despite differing evaluation goals, the introduction of these frameworks has expanded a broader concept of drug value. In the field of drug value evaluation, there is a growing trend towards incorporating a wide range of dimensions, including clinical, economic, societal and humanistic values. As proposed by Garrison LP Jr, the value of drugs cover life years gained, improvement in quality of life, cost savings within health system, productivity, cost savings outside health system, scientific spillovers, insurance value, real option value, value of hope and reduction in uncertainty (Garrison et al., 2017). Moreover, the criteria utilized to assess each dimension of value are continuously evolving. For instance, the criteria for evaluating drug efficacy have advanced from merely focusing on primary or endpoint outcomes to a comprehensive assessment of efficacy including therapeutic benefits type, multiple outcomes (primary outcome, secondary outcome, endpoint outcome, patients report outcome) and types of clinical research (Migliore et al., 2015; Vermersch et al., 2019; Institute for Quality and Efficiency in Healthcare, 2022). In situations requiring intricate decision-making, researchers tend to prioritize a holistic evaluation of drug value (Goetghebeur et al., 2012). The substantial and scattered evaluation criteria of drugs value posed inevitable challenges for the decision-making process. Collecting and categorizing these value criteria are increasingly essential to aid value-based decision-making.

Multi-Criteria Decision Analysis (MCDA), as a valuable approach for integrating diverse and complex trade-offs among different stakeholders, can provide comprehensive, transparent, and structured evaluations of drug value (Mt-Isa et al., 2014). Moreover, this approach has gained widespread use in the healthcare fields, particularly in the assessment of interventions for authorization, prioritization for coverage or reimbursement, selection for clinicians and patients, and the allocation of research funds (Marsh et al., 2014; European Medicines Agency, 2012). It is noteworthy that some prominent organizations, such as the European Medicines Agency, the Food and Drug Administration, and the International Society for Pharmacoeconomics and Outcomes Research (ISPOR), have acknowledged and endorsed MCDA as a valuable tool for assessing the benefits and safety of medicinal products (European Medicines Agency, 2012; Marsh et al., 2016; Angelis and Kanavos, 2021). Due to different evaluation purposes, various MCDA frameworks have been developed, including EVDEM (Evidence and Value: Impact on Decision Making) framework, Benefit-Risk framework, AVF (the Advance Value Framework), MAUT (Multi-attribute Utility Analysis), and others (Marsh et al., 2016). Each MCDA framework exhibits unique characteristic in selecting criteria, scoring and weighting techniques. For example, EVIDEM framework, introduced by Goetghebeur MM in 2012, employs a scoring scale ranging from 0–3 for 15 criteria (e.g., disease severity, cost-effectiveness, etc.) (Goetghebeur et al., 2012). Whereas, the benefit-risk MCDA framework utilizes a technique of “0–100 value scales” to determine the criteria score (Mendoza-Sanchez et al., 2018).

The objective of this study was to systematically collect and analyze drug value evaluation dimensions, criteria and techniques of weighting and scoring used in various MCDA frameworks based on the existing literature. This analysis aims to provide a foundation for the construction of a drug evaluation framework that could support regulatory approval, drug pricing, prescription decisions making, medical insurance reimbursement, and other related purposes.

## 2 Methods

### 2.1 Search strategies and articles selection

The related Chinese and English articles, published before 30 June 2022, had been searched at seven databases including China National Knowledge Infrastructure (CNKI), WanFang Database, the Chinese Scientific Journals Full-text Database (VIP), the Chinese Biomedical Literature Database (CBM), PubMed, Cochrane Library and Embase. We used a search strategy combining MeSH terms with free words. The search terms were composed of “MCDA” (or “multi-criteria decision analysis” or “multicriteria decision analysis” or “multicriteria decision aiding” “multi-attribute utility” or “MAU” or “MAUT” or “MACBETH” or “Evidence and Value: Impact on Decision Making” or “EVIDEM” or “Advance Value Framework”)and “intervention” (or “treatment” or “drug” or “drug assessment” or “medicine” or “medication” or “pharmacy” or “prescription”). Additional literature through other sources were also identified, such as references of relevant reviews. The detailed search strategies were described in Supplementary File S1.

Endnote X9.1 software was utilized for managing all retrieved articles. Following the removal of duplicates, two researchers (PS and KZ) independently screened articles based on predetermined inclusion and exclusion criteria. The research was limited to the application of MCDA to assess value of drug-oriented intervention and excluded the following types of studies: not in the field of medicine and health; no specific evaluation criteria; methodological or theoretical studies of MCDA; editorials; conference article and bibliographies of relevant articles.

### 2.2 Data extraction

The essential data from eligible studies were extracted using Microsoft Excel 2019 software. Two researchers (PS and KZ) independently extracted basic information using a specially designed form including publication data (publication date, title, and authors’ names, country, journal), details of MCDA (MCDA method subtypes, indication, drugs, evaluation dimensions, criteria, stakeholders, the techniques of scoring and weighting, the method of aggregation and uncertainty analysis). Any disagreement during literature screening and data extraction was resolved by consensus or consulting a third researcher (HD).

### 2.3 Descriptive statistics

The number of unique criteria and their frequencies of citation (times) in the included studies were calculated. Due to the diverse terminology used to describe similar evaluation criteria, internal deliberations were held to identify unique criteria. For example, terms referring to the same concept “comparative effectiveness/efficacy” (e.g., “overall survival period” and “progression-free survival period”) were consolidated into one criterion. Classification standard of qualitative criteria or quantitative criteria was mainly guided by the structure of the EVIDEM framework, which includes an adaptable set of qualitative criteria or quantitative criteria. Then, these unique evaluation criteria were assigned to 5 dimensions including clinical, economic, innovative, societal and humanistic value. The techniques for weighting and scoring derived from 82 articles were also counted. The greater the quantity of unique criteria, the more comprehensive the assessment perspective of these value dimensions. The more a criterion or technique were mentioned in articles, the more important we consider it.

## 3 Results

### 3.1 Literature screening

**FIGURE 1:**
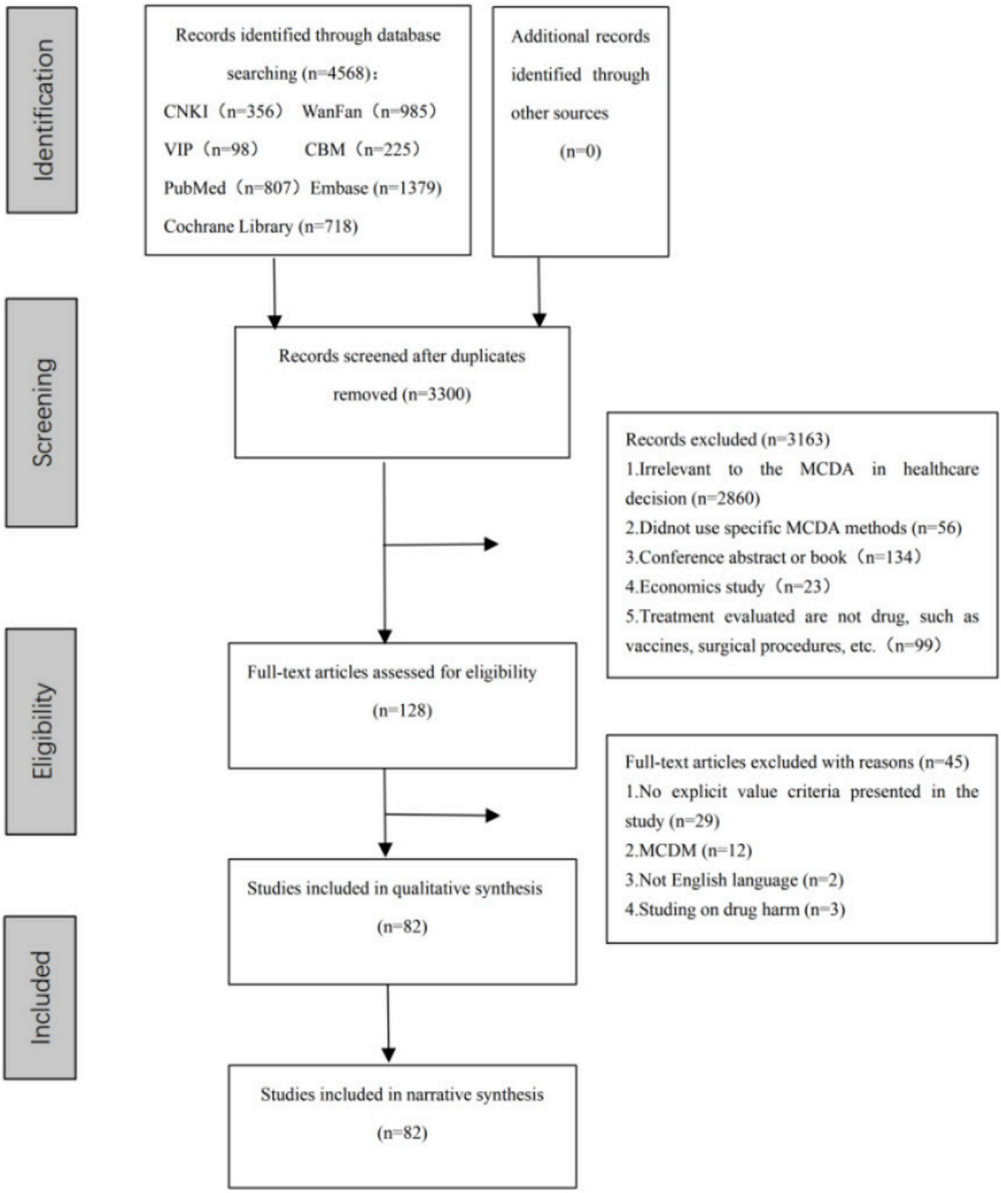
Prisma flow diagram.

Among the 4659 articles retrieved, 82 (69 in English and 13 in Chinese) articles were included in this analysis, as shown in Figure 1. There were 24 articles on EVDEM framework, 25 on Benefit-Risk Framework, 3 on AVF, 5 on MAUT, and 20 designed for specific research purposes. The first study on MCDA was published in 1991 (Schumacher, 1991), after which the number of articles showed an overall increasing trend, reaching the highest in 2016 and 2018, with 11 articles each. Spain had the highest number of articles on this subject, with a total of 17 articles. The number of criteria ranged from 4 to 38 (Sidi and Harel, 2020; Byun et al., 2016; Al-Badriyeh et al., 2016; Tervonen et al., 2015; Hsu et al., 2015; Erjaee et al., 2012; Bettinger et al., 2007; Pérez Encinas et al., 1998). Additional information regarding the literature included can be accessed in Supplementary File S2.

### 3.2 Evaluation dimensions and criteria

**FIGURE 2:**
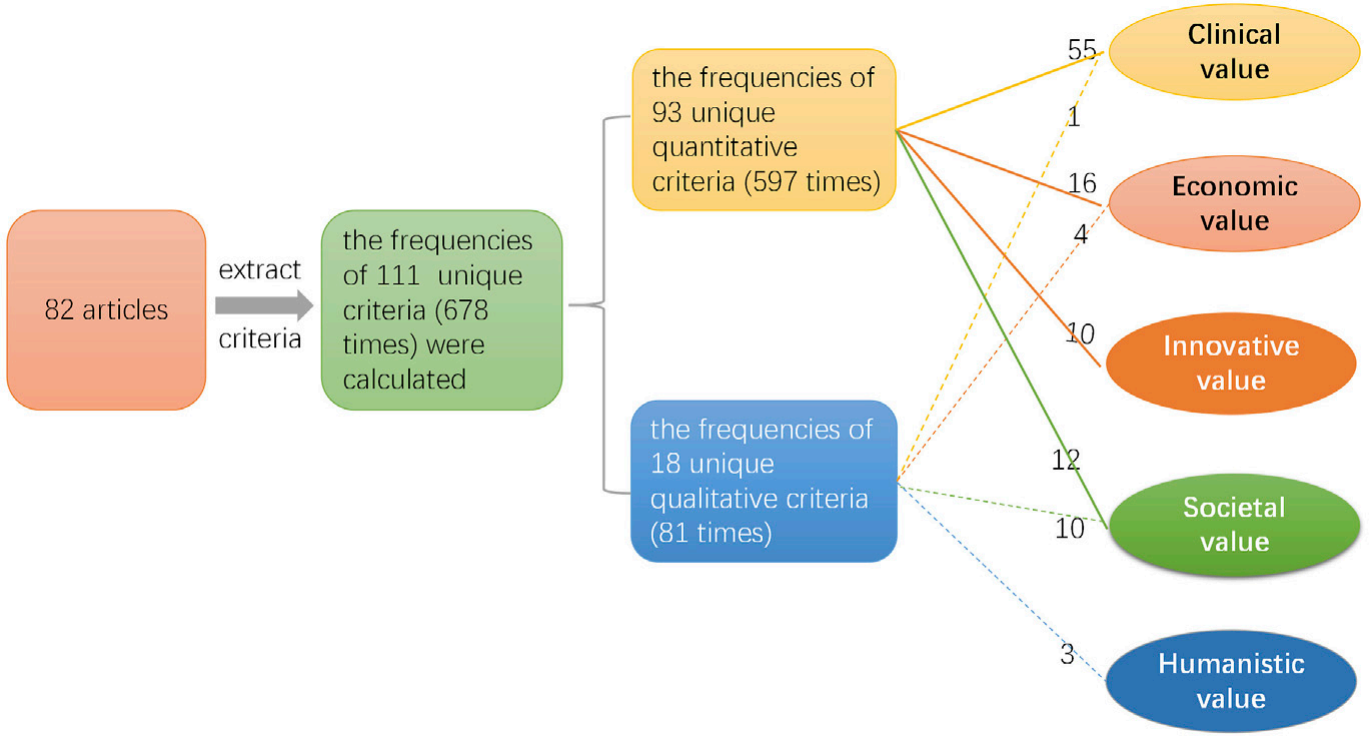
Research diagram of each dimension criteria.

Drug value evaluation covered 5 dimensions (clinical, economic, societal, humanistic, and innovative value) and 111 unique criteria (678 times) in the 82 included articles. These criteria could be divided into quantitative criteria and qualitative criteria, as shown in Figure 2.

#### 3.2.1 Clinical value

**FIGURE 3:**
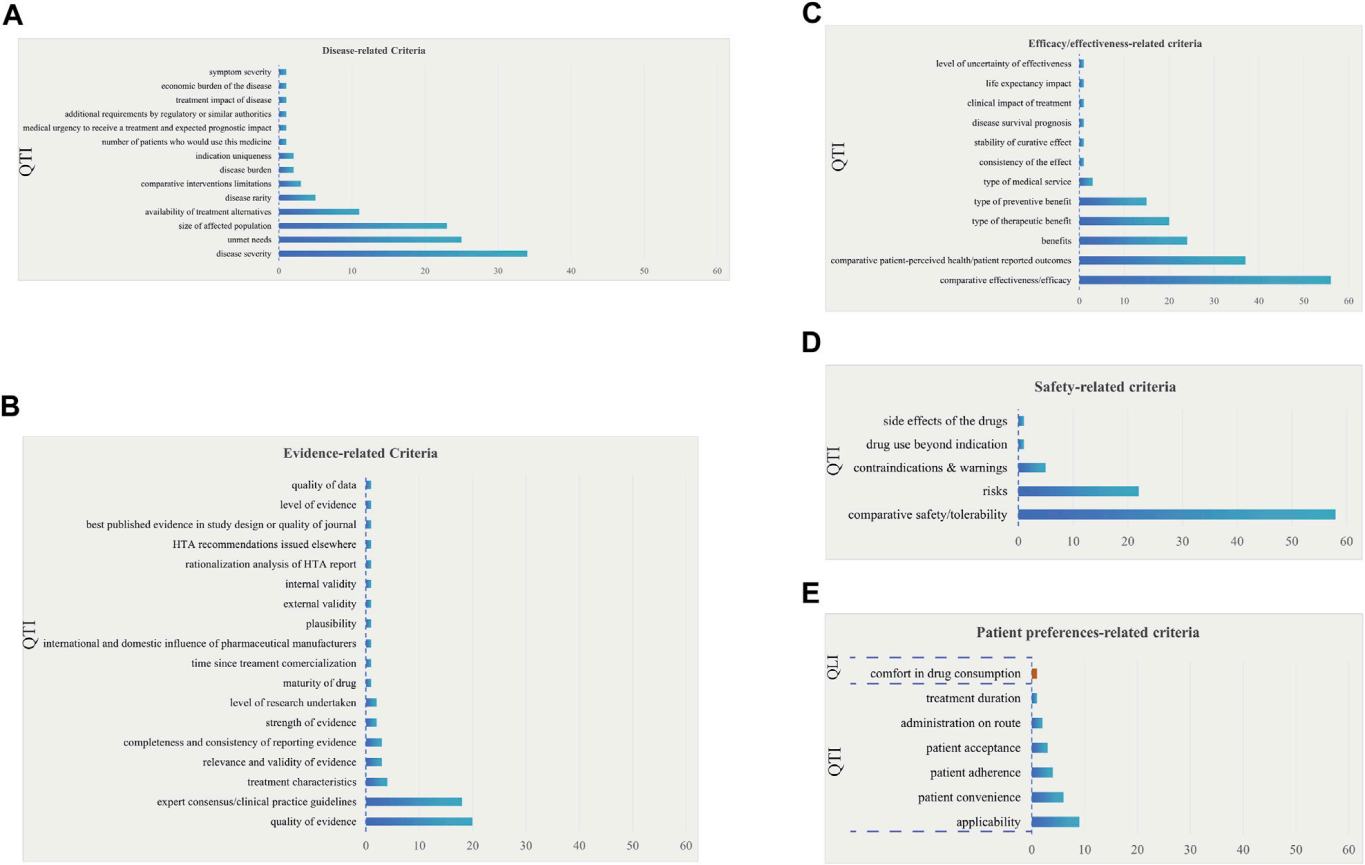
The frequency plot of clinical value criteria occurrence. Note: Clinical value criteria were further categorized into five groups: disease-related criteria **(A)**, evidence-related criteria **(B)**, effectiveness/efficacy-related criteria **(C)**, safety-related criteria **(D)** and patient preferences-related criteria **(E)**. The blue bars represent quantitative criteria, while orange bars represent qualitative criteria. QTI stands for “quantitative” and QLI stands for “qualitative.”

This study identified 56 unique clinical value criteria (448 times), with the criterion of “comfort in drug consumption” being the sole qualitative criterion, as shown in Figure 3. These criteria were further categorized into five groups: disease-related criteria, evidence-related criteria, effectiveness/efficacy-related criteria, safety-related criteria and patient preferences-related criteria.

##### 3.2.1.1 Disease-related criteria

In the analysis of 14 unique disease-related criteria (111 times) (Figure 3A), the most frequently mentioned were “disease severity” (34 times), “unmet needs” (25 times), and “size of affected population” (23 times). It is worth noting that the criterion of “the size of the population being affected by the disease” was not recommended by AVF due to ethical concerns related to evaluating the clinical value of drug based on high or low disease prevalence (Angelis and Kanavos, 2017). “Disease rarity” could be used as a supplementary criterion to assess the degree of rarity of the disease.

##### 3.2.1.2 Evidence-related criteria

Within the 18 unique evidence-related criteria (63 times) (Figure 3B), the two criteria mentioned over 10 times were “quality of evidence” (20 times) and “expert consensus/clinical practice guidelines” (18 times).

##### 3.2.1.3 Effectiveness/efficacy-related criteria

Among the 12 unique effectiveness/efficacy-related criteria (161 times) (Figure 3C), the five criteria that were cited more than 10 times included “comparative effectiveness/efficacy” (56 times), “comparative patient-perceived health/patient reported outcomes” (37 times), “benefits” (24 times), “type of therapeutic benefit” (20 times), and “type of preventive benefit” (15 times).

##### 3.2.1.4 Safety-related criteria

Within the 5 unique safety-related criteria (87 times) (Figure 3D), the two criteria mentioned over 10 times were “comparative safety/tolerability” (58 times) and “risks” (22 times).

##### 3.2.1.5 Patient preference-related criteria

Among the 7 unique criteria related to patient preferences (26 times) (Figure 3E), the top three most frequently mentioned were “applicability” (9 times), “patient convenience” (6 times), and “patient adherence” (4 times).

#### 3.2.2 Economic value

**FIGURE 4:**
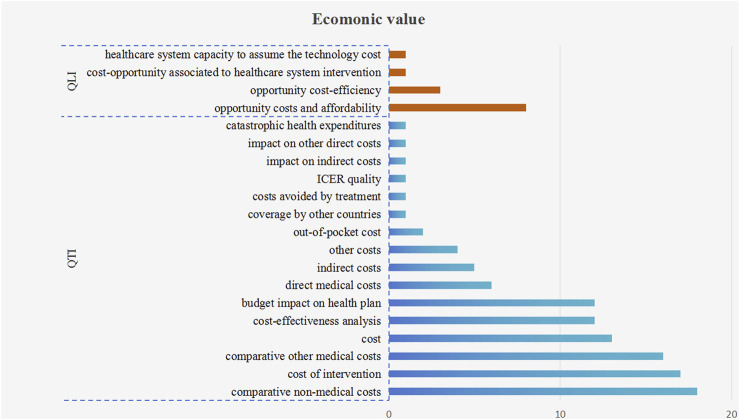
The frequency plot of economic value criteria occurrence. Note: The blue bars represent quantitative criteria, while orange bars represent qualitative criteria. QTI stands for “quantitative” and QLI stands for “qualitative.”

Among the 16 quantitative criteria (111 times) and 4 qualitative criteria (13 times) which were employed to assess the economic value, the most frequently utilized top three criteria were “cost of intervention” (17 times), “comparative other medical costs” (16 times), and “comparative non-medical costs” (18 times), as shown in Figure 4. In certain instances, comprehensive economic indicators, such as “budget impact on health plan” (12 times) and “cost-effectiveness analysis” (12 times) were also employed for economic value assessment. Furthermore, the AVF frameworks employed medical costs impact, rather than intervention cost, to assess the socioeconomic impact. Additionally, the qualitative criteria of “opportunity costs and affordability” (8 times), “opportunity cost-efficiency” (3 times), “healthcare system capacity to assume the technology cost” (1 time), “cost-opportunity associated to healthcare system intervention” (1 time) were also utilized to assess the economic value.

#### 3.2.3 Innovative value

**FIGURE 5:**
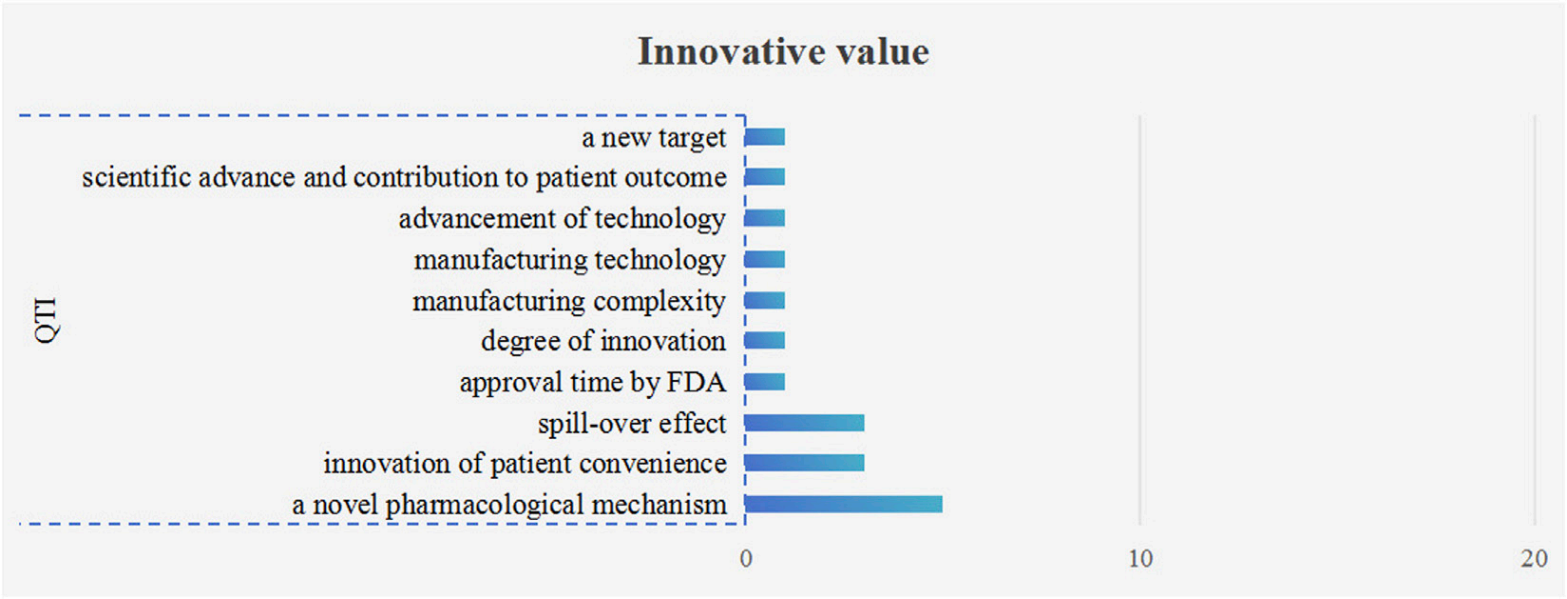
The frequency plot of innovative value criteria occurrence. Note: The blue bars represent quantitative criteria QTI stands for “quantitative.”

There were 10 quantitative criteria (18 times) used to assess the innovation of drugs, as shown in Figure 5. Among these criteria, the most frequent occurrence was “a novel pharmacological mechanism” (5 times). The criteria of “spill-over effect” and “innovation of patient convenience” were mentioned 3 times each.

#### 3.2.4 Societal value

**FIGURE 6:**
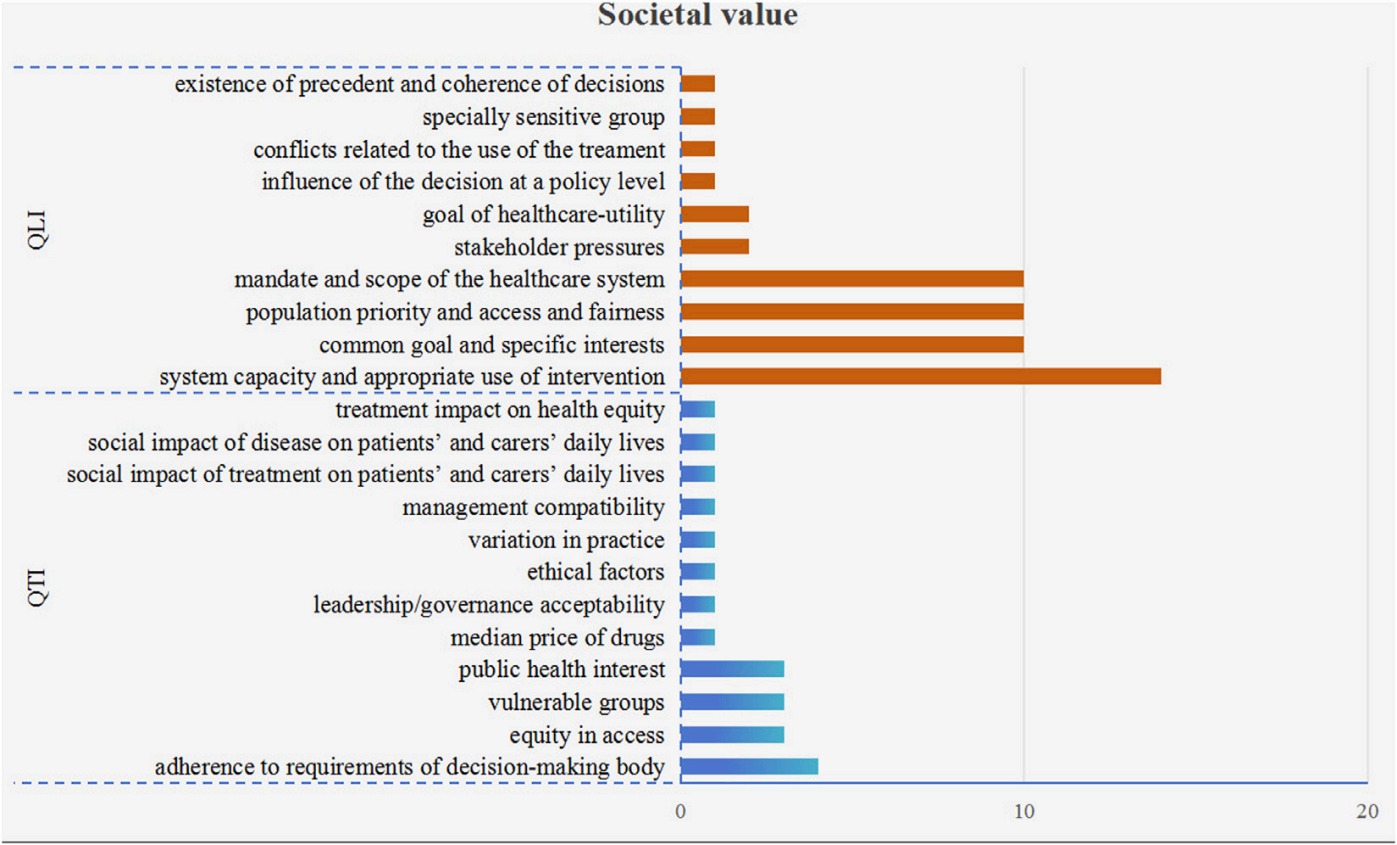
The frequency plot of societal value criteria occurrence. Note: The blue bars represent quantitative criteria, while orange bars represent qualitative criteria. QTI stands for “quantitative” and QLI stands for “qualitative.”

10 quantitative (52 times) and 12 qualitative criteria (21 times) were used to measure societal value, as shown in Figure 6. Among these criteria, there were four criteria mentioned over 10 times, namely, “system capacity and appropriate use of intervention” (14 times), “common goal and specific interests” (10 times), “mandate and scope of the healthcare system” (10 times), and “population priorities and access and fairness” (10 times). It is also worth noting that all of these four criteria were qualitative.

#### 3.2.5 Humanistic value

**FIGURE 7:**
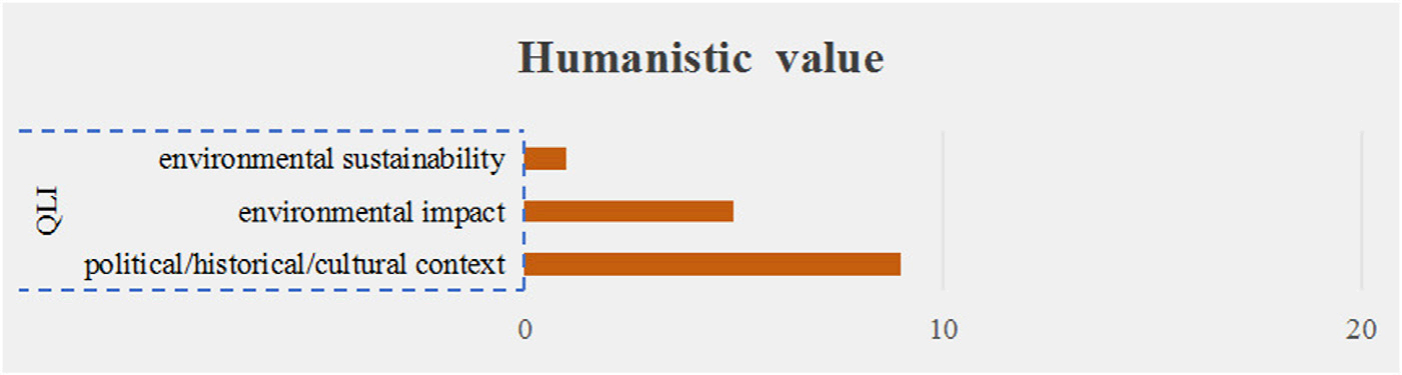
The frequency plot of humanistic value criteria occurrence. Note: The orange bars represent qualitative criteria. QLI stands for “qualitative.”

Out of 3 qualitative criteria (15 times) used to measure the humanistic value of drugs, “political/historical/cultural context” was the most frequently mentioned criterion (9 times), followed by “environmental impact” (5 times) and “environmental sustainability” (1 time), as shown in Figure 7.

### 3.3 The techniques for weighting

**FIGURE 8:**
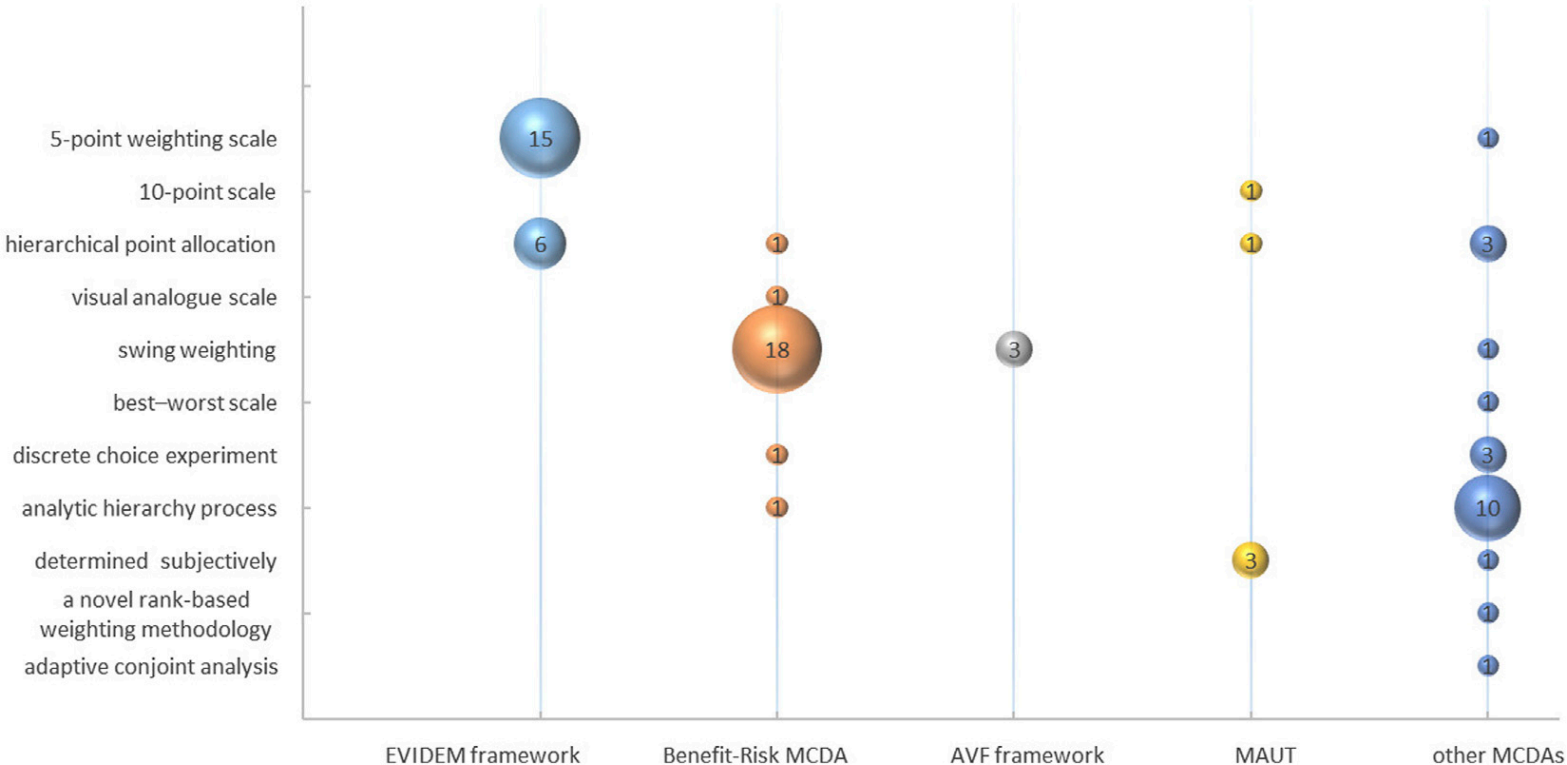
The techniques for weighting.

11 unique techniques for weighting were identified, including “5-point weighting scale,” “10-point scale,” “Hierarchical Point Allocation (HPA),” “Visual Analogue Scale (VAS),” “Swing Weighting,” “best-worst scale,” “Discrete Choice Experiment (DCE),” “Analytic Hierarchy Process (AHP),” “determined subjectively,” “a novel rank-based weighting methodology” and “Adaptive Conjoint Analysis (ACA).” “Swing Weighting” was the most commonly utilized technique among articles, as illustrated in Figure 8.

Moreover, the application of weighting techniques exhibited variability among various MCDA frameworks. Within benefit-risk MCDA studies, 81.82% (18/22) adopted “Swing Weighting.” All the AVF Framework studies (3/3) utilized “Swing Weighting” to determine the criteria weights. In MUAT studies, researchers predominantly determined criteria weights through a subjective process, combining the level of criteria and evidence from the literature (3/5). Other MCDA studies with specific research objectives utilized AHP (10/22) to weight criteria.

### 3.4 The technique for scoring

**FIGURE 9:**
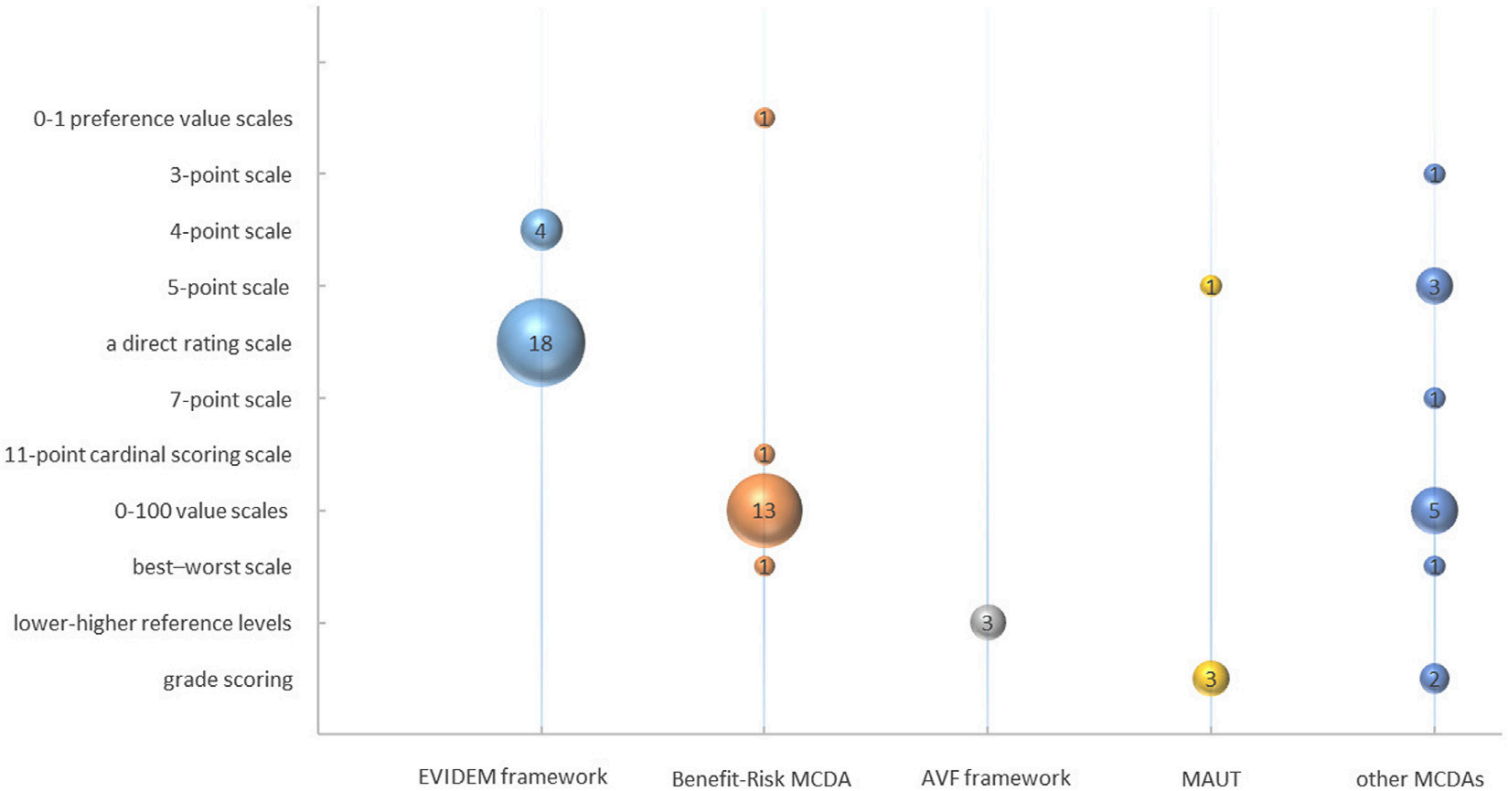
The technique for scoring.

11 techniques were utilized for estimating scoring, including “0–1 preference value scales,” “3-point scale,” “4-point scale,” “5-point scale,” “a direct rating scale,” “7-point scale,” “11-point cardinal scoring scale,” “0–100 value scales,” “best-worst scale,” “lower-higher reference levels,” and “grade scoring.” “A direct rating scale” was the most commonly employed, being utilized in 18 of the articles analyzed, as depicted in Figure 9. This technique primarily measured the criteria scoring based on the “performance matrix” of each intervention through expert meetings. Alternatively, researchers scored directly based on the literature.

In the EVIDEM framework studies, 81.82% (18/22) employed “a direct rating scale,” while the others (4/22) used a “4-point scale.” Among the benefit-risk MCDA framework studies, 81.25% (13/16) utilized “0–100 value scales” to measure scores of criteria. The AVF framework adopted “lower-higher reference levels” to establish the range of criteria scores (3/3). In MAUT studies, criteria scoring was measured through grade scoring (3/4) and a 5-point scale (1/4). The grade levels for each criterion were predetermined and subsequently utilized by researchers to assign corresponding scores after conducting a thorough review of the literature. Some MCDA studies with specific research purposes adopted more flexible methods of scoring, such as 0–100 value scales (5/13), grade scoring (2/13), 5-point scales (2/13), or other techniques (4/13).

## 4 Discussion

Our analysis of 82 literature revealed that the evaluation of drug values covered 5 dimensions and 111 criteria (678 times). 94 (94/111, 84.68%) criteria were quantitative. Of these, 56 of the 94 criteria (59.57%) measured clinical value. Furthermore, we identified 11 scoring and 11 weighting techniques used in various MCDA frameworks. “Swing weighting” and “a direct rating scale” were the most frequently techniques used in MCDA literature. To the best of our knowledge, our study was the first attempt to categorize drug-oriented MCDA criteria based on their clinical, economic, societal, innovative and humanistic values.

The evaluation of drug value is a multifaceted process that requires thorough consideration of various dimensions, such as medical, ethical, economic, and social equity criteria (Garrison et al., 2017; Lakdawalla et al., 2018). Therefore, it is imperative to categorize the criteria for drug evaluation based on different value dimensions. However, previous literature reviews on MCDAs in healthcare have exhibited certain limitations, including oversimplified categorization of criteria. Lalla Aida Guindo classified the criteria based on the evaluation dimensions of EVIDEM framework (Guindo et al., 2012), while Tamas Zelei sorted out the decision criteria based on cost and outcome dimensions (Zelei et al., 2021). Additionally, a wider range of interventions was encompassed, such as pharmaceuticals, public health interventions (e.g., smoking cessation, obesity), screening, surgical strategies, devices, (Marsh et al., 2014). Further detailed research is required to explore the unique characteristics of different interventions in value evaluation. The National Institute for Health and Care Excellence (NICE) utilizes topic classification to develop health technology assessment, encompassing devices, diagnostics, interventional procedures, medicines, combination or integrated technologies, digital technologies and other technologies (National Institute for Health and Care Excellence, 2022). Our research focused on pharmaceuticals and categorized for drug-oriented MCDA criteria according to 5 dimensions (clinical, economic, societal, innovative and humanistic value dimensions). We found that clinical value criteria were commonly utilized, followed by economic value criteria, which aligns with previous studies (Park et al., 2012; Kwon et al., 2017; Tanios et al., 2013; Golan et al., 2011; Baji et al., 2016). Clinical value is the core factor in drug development and use, which can reflect the extent of a drug’s ability to meet clinical needs and provide clinical benefits for its target population (Liu et al., 2021).

In relation to clinical value, the regulatory and reimbursement processes for drugs prioritize the assessment of the effectiveness-safety balance, also referred to as efficacy-tolerability or benefit-risk balance (Dang et al., 2020). For example, the Food and Drug Administration (FDA) conducted a thorough benefit-risk evaluation of new drugs, taking into account the substantial evidence of safety and effectiveness provided by the sponsor (Food and Drug Administration, 2021). Similarly, the Institute for Quality and Efficiency in Healthcare (IQWiG) in Germany made a trade-off between benefit and harm aspect in the early benefit assessment of new drugs, and determined the extent of added benefit (minor, considerable, and major treatment effects) (Institute for Quality and Efficiency in Health Care, 2022). Our study also confirmed that “comparative safety/tolerability” was the most frequently used clinical value criterion, followed by “comparative effectiveness/efficacy.” Although there are subtle differences between efficacy and effectiveness, as well as safety and tolerability (Berger et al., 2012), we aggregated “effectiveness” and “efficacy” criteria into a single “comparative effectiveness/efficacy” criterion considering greater operability in practice, as well as “comparative safety/tolerability.”

With regard to the economic value, we found the most frequently utilized criteria were “cost of intervention,” “comparative other medical costs” and “comparative non-medical costs.” However, it is constantly discussing whether to incorporate cost (and related criteria such as cost-effectiveness, budget impact) as a criterion in MCDA (Golan et al., 2011; Marsh et al., 2018a; Hansen and Ombler, 2008). Hansen argued “cost is not a value criterion, but a measure of what has to be given up to achieve the value criteria” (Hansen and Devlin, 2019). Including cost related criteria in the calculation of MCDA values may violate the principle of “structurally independent” of the multidimensionality of value and damage opportunity cost in the allocation of limited resources (Hansen and Devlin, 2019; Wilson et al., 2022). Wilson compared the extent of consistency in ranking importance of the four criteria of “treatment effectiveness,” “cost of the intervention” “risk of serious harms,” and “risk of mild-to-moderate harms” based on MCDA and cost-effectiveness analysis models and found “cost of the intervention” should be excluded in MCDA for prioritizing intervention setting (Wilson et al., 2022). Golan and Angelis also excluded the cost of the treatments in the MCDA value metric. Golan used MCDA to aggregate health-related benefits of multiple dimensions and calculated the final value score by formula “cost/health-related benefits value” (Golan and Hansen, 2012). Angelis used costs per unit of MCDA value to calculate overall weighted preference value scores (i.e., the final value scores) (Angelis et al., 2020). However, cost related criteria were incorporated in EVIDEM frameworks (Goetghebeur et al., 2012). A set of criteria including “direct medical costs,” “direct non-medical costs” and “indirect costs” were used as the “modulators” of core model to measure economic consequences of intervention (Casellas Caro et al., 2022). With the soaring drug costs and limited financial resources, we think cost of the interventions should be considered in decision making, especially in prioritizing drugs for reimbursement.

Medical innovation is a complex concept lacking a definitive consensus, which mainly related to the realm of “therapeutic innovation” (Tanios et al., 2013). Therapeutic innovation is commonly associated with generating improved health outcomes that were previously unattainable and addressing the unmet medical need (Morgan et al., 2008). Nevertheless, we found the AVF frameworks offer a distinct viewpoint for quantitatively assessing the value of innovation, utilizing criteria such as the mechanism of action of the medicine, its spill-over effects, and its utility for patients (e.g., convenience) (Angelis and Phillips, 2021). The criterion of “mechanism of action” can be evaluated by Anatomical Therapeutic Chemical (ATC) Classification System of World Health Organization (WHO). The degree of innovation “spill-over effects,” as argued by Angelis, can be gauged by the number of new indications being explored for the medicine at each stage of clinical development (e.g., Phase I, Phase II, Phase III, Marketing Authorization phase). “Patient usefulness” could be perceived as ease and convenience, which were related to mode of administration, dosing schedule, medication restrictions, and product-specific designs.

Once the criteria are agreed upon in MCDA frameworks, the selection of the appropriate techniques of scoring and weighting are subsequently considered by the researchers. A published study in healthcare found AHP is the most frequently applied technique for weighting, followed by “Swing Weighting” (Camps et al., 2019). However, our research showed that “Swing Weighting” and “a direct rating scale” were the most frequently techniques used in the field of pharmaceuticals assessment. “Swing Weighting” could obtain more discriminative weights by obtaining the expected performance floating range of each criterion in advance. It should be noted that the weight of each criterion is typically determined by a panel of experts, independent of the specific drug (Hansen and Devlin, 2019; Camps et al., 2020; Guarga et al., 2019; Jiménez et al., 2018). Due to the weights and scores were closely related to the panel of experts participating in MCDA, the process of weight and score determination were subjective to some extent. So, sensitivity analysis was often used to assess the robustness of MCDA results by re-testing using alternative weighting and scoring techniques. Furthermore, as Marsh argued, it is important to incorporate well-trained experts and working groups to perform the MCDA (Marsh et al., 2018b).

Some limitations were worth mentioning. Firstly, the identified literature was only searched in seven publicly available databases, and supplemented with relevant review references and expert consultations. Grey articles were not acquired in this study, which may lead to a publication bias to a certain extent. Secondly, due to language limitations, only Chinese and English literature were identified. Excluding articles published in other languages may have had an impact on our results. Thirdly, this study did not recognize the critical dimensions and criteria targeted for different stages of life cycles in drug value evaluation. In future, we will continue to carry out the research on drug value assessment in different stages of life cycles.

## 5 Conclusion

This study comprehensively presented the current evaluation dimensions, criteria, and techniques for scoring and weighting in drug-oriented MCDA articles. By highlighting the frequently cited evaluation criteria and techniques for scoring and weighting, this analysis can serve as a resource to reasonably select these evaluation details in constructing the appropriate MCDA framework. The ultimate objective is to provide a solid foundation for the construction of a drug evaluation framework to advancing the structured decision in drug management. In future, as research on MCDA drug value evaluation deepens, more attention should be paid to the assessment criteria and techniques of weighting and scoring for different types of drugs. For instance, specific criteria should be considered when assessing traditional Chinese medicine in China.

## Abbreviations

ACA, adaptive conjoint analysis; AHP, analytic hierarchy process; ATC, anatomical therapeutic chemical; AVF, advance value framework; DCE, discrete choice experiment; EVDEM, evidence and value: impact on decision making; FDA, food and drug administration; HPA, hierarchical point allocation; HTA, health technology assessment; IQWiG, institute for quality and efficiency in healthcare; ISPOR, international society for pharmacoeconomics and outcomes research; MAUT, multi-attribute utility analysis; MCDA, multi-criteria decision analysis; NICE, national institute for health and care excellence; VAS, visual analogue scale; WHO, world health organization.
