# Supplementary material for: The application of multi-criteria decision analysis in evaluating the value of drug-oriented intervention: a literature review
Source: Front Pharmacol. 2024 Apr 24;15:1245825. doi: 10.3389/fphar.2024.1245825 (PMC11076741; doi:10.3389/fphar.2024.1245825)
Supplement: Supplementary file 1 [file DataSheet1.PDF]

Additional File 1: the search strategies

We searched four Chinese databases and three English databases.

1.China National Knowledge Infrastructure (CNKI)

TKA= 'MCDA' or TKA= 'MCDM' or TKA= '多准则决策分析' or TKA= '多准则决策' or TKA= '多属性效用' or TKA= '多属性效用理论' or TKA= 'MACBETH' or TKA= 'EVIDEM' or TKA= '优化价值框架'

Search restriction "医药卫生科技领域"

2.WanFang Database

(资源类型=(学位论文 OR 中文期刊 OR 会议论文)

((((((摘要="MCDA") OR 摘要="MCDM") OR 摘要=多准则决策) OR 摘要=多属性效用) OR 摘要="MACBETH") OR 摘要="EVIDEM") OR 摘要="优化价值框架"

3. the Chinese Scientific Journals Full-text Database (VIP)

M=(MCDA OR MCDM OR 多准则决策分析 OR 多准则决策 OR 多属性效用 OR 多属性效用理论 OR MACBETH OR EVIDEM OR 优化价值框架)

Search restriction "医药卫生领域"

4. the Chinese Biomedical Literature Database (CBM)

"MCDA"[摘要:智能] OR "MCDM"[摘要:智能] OR "多准则决策分析"[摘要:智能] OR "优化价值框架"[摘要:智能] OR "EVIDEM"[摘要:智能] OR "MACBETH"[摘要:智能] OR "多属性效用理论"[摘要:智能] OR "多属性效用"[摘要:智能] OR "多准则决策"[摘要:智能]

5. Pubmed

#1:

"MCDA"[Title/Abstract] OR "MCDM"[Title/Abstract] OR "multi-criteria decision analysis"[Title/Abstract] OR "multi-criteria decision making"[Title/Abstract] OR "multicriteria decision analysis"[Title/Abstract] OR "multicriteria decision aiding"[Title/Abstract] OR "multicriteria decision making"[Title/Abstract] OR "multiple criteria decision analysis"[Title/Abstract] OR "multiple criteria decision aiding"[Title/Abstract] OR "multiple criteria decision making"[Title/Abstract] OR "multiattribute utility"[Title/Abstract] OR "MAU"[Title/Abstract] OR "multiattribute utility theory"[Title/Abstract] OR "MAUT"[Title/Abstract] OR "MACBETH"[Title/Abstract] OR "Evidence and Value: Impact on Decision Making"[Title/Abstract] OR "EVIDEM"[Title/Abstract] OR "Advance Value Framework"[Title/Abstract]

#2:

"intervention"[Title/Abstract] OR "treatment"[Title/Abstract] OR "drug"[Title/Abstract] OR "drug assessment"[Title/Abstract] OR "medicine"[Title/Abstract] OR "medication"[Title/Abstract] OR "pharmacy"[Title/Abstract] OR "prescription"[Title/Abstract]

#3: #1 and #2

6. Embase

('mcda':ab,ti OR 'mcdm':ab,ti OR 'multi-criteria decision analysis':ab,ti OR 'multi-criteria decision

aiding':ab,ti OR 'multi-criteria decision making':ab,ti OR 'multicriteria decision analysis':ab,ti OR 'multicriteria decision aiding':ab,ti OR 'multicriteria decision making':ab,ti OR 'multiple criteria decision analysis':ab,ti OR 'multiple criteria decision aiding':ab,ti OR 'multiple criteria decision making':ab,ti OR 'multicriteria analysis':ab,ti OR 'multiattribute utility':ab,ti OR 'mau':ab,ti OR 'multiattribute utility theory':ab,ti OR 'maut':ab,ti OR 'measuring attractiveness by a categorical based evaluation technique':ab,ti OR 'macbeth':ab,ti OR 'evidence and value: impact on decision making':ab,ti OR 'evidem':ab,ti OR 'advance value framework':ab,ti) AND ('intervention':ab,ti OR 'treatment':ab,ti OR 'drug':ab,ti OR 'drug assessment':ab,ti OR 'medicine':ab,ti OR 'medication':ab,ti OR 'pharmacy':ab,ti OR 'prescription':ab,ti)

7. Cochrane library
#1:
(MCDA):ti,ab,kw OR (MCDM):ti,ab,kw OR (multi-criteria decision analysis):ti,ab,kw OR (multi-criteria decision making):ti,ab,kw OR (multicriteria decision analysis):ti,ab,kw OR (multicriteria decision aiding):ti,ab,kw OR (multicriteria decision making):ti,ab,kw OR (multiple criteria decision analysis):ti,ab,kw OR (multiple criteria decision aiding):ti,ab,kw OR (multiple criteria decision making):ti,ab,kw OR (multiattribute utility):ti,ab,kw OR (MAU):ti,ab,kw OR (multiattribute utility theory):ti,ab,kw OR (MAUT):ti,ab,kw OR (MACBETH):ti,ab,kw OR (Evidence and Value Impact on Decision Making):ti,ab,kw OR (EVIDEM):ti,ab,kw OR (Advance Value Framework):ti,ab,kw
#2:
(intervention):ti,ab,kw OR (treatment):ti,ab,kw OR (drug):ti,ab,kw OR (drug assessment):ti,ab,kw OR (medicine):ti,ab,kw OR (medication):ti,ab,kw OR (pharmacy):ti,ab,kw OR (prescription):ti,ab,kw
#3: #1 and #2

Additional File 2: the included literature

Study ID	Country	MCDA subtypes	Diseases	Intervention	Criteria numbers	stakeholders	Expert numbers
Zozaya, N 2022 (1)	Spain	EVIDEM	hereditary angioedema	lanadelumab, C1-inhibitor, danazol， placebo	13	patients, physicians, pharmacists, decision-makers	10
Zozaya, N 2022 (2)	Spain	EVIDEM	moderate-to-severe psoriasis plaque psoriasis	bimekizumab, adalimumab, ustekinumab, secukinumab, ixekizumab, risankizumab, placebo	15	clinicians, nurse, psychologist, patients, healthcare managers, hospital pharmacist, health economist, representative of the political sphere	12
Gil-Nagel, A. 2022 (3)	Spain	EVIDEM	dravet Syndrome	fenfluramine, cannabidiol, clobazam, stiripentol, topiramate	13	neurologists, active participation in clinical trials, hospital pharmacists, parents of patients, former national and regional decision-makers	10
Gasol, M. 2022 (4)	Spain	EVIDEM	severe or rare diseases	--	12	evaluators, policymakers, clinicians, policymakers, patient representatives	18
Casellas Caro, M. 2022 (5)	Spain	EVIDEM	iron deficiency anaemia	ferric carboxymaltose, ferrous sulphate	25	Gynaecologists, obstetricians,haematologists, anaesthesiologists, midwives, hospital pharmacists, decision-makers, patients representative	12
Yuhan D 2022 (6)	China	EVIDEM	--	voronosan fumarate tablets and eprazole enteric-coated tablets	13	--	--

Chuqiao Y 2022 (7)	China	Benefit-risk	chronic hepatitis B hepatic fibrosis	entecavir combined with Fuzheng Huayu Capsules, Fufang Biejia Ruangan Tablets, Anluo Huaxian Pills	10	--	--
Vandewalle, B. 2021 (8)	Portugal	Other MCDAs	paediatric X-linked hypophosphatemia	burosumab, conventional therapy	14	physicians, medical geneticist, patient representatives, health economist, health policy maker	9
Karrer, L. 2021 (9)	German	EVIDEM	thyroid nodules	--	17	Physicians, patients	79
Bao, Y. 2021 (10)	China	EVIDEM	--	sitagliptin, linagliptin, vildagliptin, alogliptin, saxagliptin	19	doctors, nurse, pharmacist, health economist, decision-maker	6
Nutt, D. J. 2021 （11）	UK	Benefit-risk	chronic neuropathic pain	tetrahydrocannabinol (THC)/ cannabidiol 1:1, CBD dominant, THC dominant, Duloxetine, Gabapentinoids, Amitriptyline, Tramadol, Ibuprofen, Methadone, Oxycodone, Morphine, Fentanyl	17	clinicians (pain specialists and psychiatrists), scientists, and patient representatives	--
Milsom, I. 2021 (12)	Sweden	Benefit-risk	oveactive bladder (OAB)	fesoterodine 4 or 8, solifenacin 5/mirabegron 50, solifenacin 5/mirabegron 25, solifenacin 10, trospium 20, fesoterodine8, solifenacin5, tolterodine4, mirabegron 50, fesoterodine4, mirabegron 25, placebo, darifenacin15, oxybutynin10,	11	patients, physicians	--

				darifenacin7.5			
Kremer, I. E. H. 2021 (13)	Netherlands	Other MCDAs	relapsing-remitting multiple sclerosis	interferon beta-1b, interferon beta-1a IM, interferon beta-1a SC, peginterferon beta-1a, glatiramer acetate, teriflunomide, dimethyl fumarate, natalizumab, alemtuzumab, fingolimod, cladribine, ocrelizumab	27	Patients, healthcare professionals, nurses, an expert in patient decision aid development	19
Athanasakis, K. 2021 (14)	Greece	Other MCDAs	rheumatoid arthritis	abatacept, adalimumab, certolizumab pegol, etanercept, golimumab, infliximab, rituximab, tocilizumab, tofacitinib	8	Physicians, patients	4
de Andrés-Nogales, F. 2021 (15)	Spain	Other MCDAs	rare diseases	orphan medicinal products	13	physicians, hospital pharmacists, health economists, patient representatives, members from national and regional health authorities	28
Angelis, A. 2021 (16)	UK	Benefit-risk	systemic lupus erythematosus	belimumab 10 mg, belimumab 1 mg, placebo	9	clinical assessors, non-clinical assessor, quality assessor, pharmacist	5
Qianqian H 2021 (17)	China	Benefit-risk	rheumatoid arthritis	Chinese materia medica compound preparation of Strychni Semen alone or in combination with conventional chemicals	13	--	--
Hao J 2021 (18)	China	Benefit-risk	primary hepatic carcinoma	aidi injection, aiyishu injection	13	--	--

Camps, C. 2020 (19)	Spain	EVIDEM	breast cancer	abemaciclib	8	oncologists	9
Sidi, Y. 2020 (20)	USA	Benefit-risk	--	noninferiority treatment, standard of care	4	--	--
Schug, S. 2020 (21)	Australia	Benefit-risk	postoperative Pain	acetaminophen, diclofenac, ketorolac, metamizole, morphine, nefopam, parecoxib, tramadol	17	clinicians, expert in MCDA, industry-based experts, industry-based researcher	7
de Andrés-Nogales, F. 2020 (22)	Spain	Other MCDAs	diabetic macular edema	--	10	physicians, pharmacists, health authorities, health management experts, psychologist, patient representatives	19
Babashov, V. 2020 (23)	Canada	Other MCDAs	antitumor drugs	--	7	--	--
Angelis, A. 2020 (24)	Sweden、Andalusia、Poland、Belgium	AVF	--	enzalutamide, abiraterone, cabazitaxel	18	--	--
Angelis, A. 2020 (25)	England, France, Germany	AVF	nonalcoholic fatty liver disease	publicly available product, hypothetical product	14, 16, 17	specifically, health care professionals, methodologists, patient representatives	51
Lin W 2020 (26)	China	Benefit-risk	depressive disorder	Vortioxetine 2.5，5，10，15，20 $mg \cdot d^{-1}$	20	--	--
Hao J 2020 (27)	China	Benefit-risk	rheumatoid arthritis	tripterygium wilfordii polyglycoside tablets alone and combined with immunosuppressive drugs	16	--	--

Wudong G 2020 (28)	China	Other MCDAs	tumour	antitumor drug	18	--	--
Guarga L. 2019 (29)	Spain	EVIDEM	rare diseases	--	15	clinicians, hospital pharmacists, decision-makers, patient representatives	7
Guarga, L. 2019 (30)	Spain	EVIDEM	rare diseases	--	14	physicians, healthcare service managers, pharmacists, patient representative	18
Baran-Kooiker, A. 2019 (31)	Kazakhstan, Netherlands, Poland, Romania, Russia, Turkey, Ukraine	EVIDEM	rare diseases	--	15	doctors, pharmacists, lawyers, economists, biologists, chemists, biotechnologists, others working for governmental organizations, universities, hospitals/clinics, pharmaceutical/consulting companies, patients.	140
Badia, X. 2019 (32)	Europe	EVIDEM	--	hypothetical oncologic treatment	16	general patient, general cancer patient, patients with a specific cancer type	8
Vermersch, P. 2019 (33)	France	Benefit-risk	relapsing-remitting multiple sclerosis	cladribine, alemtuzumab, dimethyl fumarate, fingolimod, natalizumab, teriflunomide	18	neurologists	--
Hsu, J. C. 2019 (34)	Taiwan	Other MCDAs	metastatic colorectal cancer	bevacizumab, cetuximab, panitumumab, aflibercept, regorafenib	9	National Health Insurance Administration, Food and Drug Administration, Experts/Scholars, Patients Group, Physicians, Pharmacists,	30

						Hospitals, Industrialists	
Mendoza-Sanchez, J. 2018 (35)	Canada	Benefit-risk	atrial fibrillation	apixaban, dabigatran, warfarin, rivaroxaban	7	cardiologist, internist and vascular neurologist	3
Zozaya, N. 2018 (36)	Spain	EVIDEM	chronic inflammatory skin diseases	dupilumab, secukinumab	13	clinicians, patients, regional payers, health economist	10
Jiménez, A. 2018 (37)	Spain	EVIDEM	pulmonary arterial hypertension	selexipag, inhaled iloprost	16	evaluators, clinicians, regional decision makers, hospital pharmacists, patients	28
Roldán, U. B. 2018 (38)	Spain	EVIDEM	gaucher disease、inflammatory bowel disease	orphan drug	9	hospital pharmacists, clinicians, clinicians, medical director	11
Wagner, M. 2018 (39)	Spain	EVIDEM	nonfunctioning gastroenteropancreatic neuroendocrine tumors	lanreotide, watchful waiting	31	Patients, physicians	11
Wagner, M. 2018 (40)	the United States	EVIDEM	gastroenteropancreatic neuroendocrine tumors	watchful waiting, somatostatin analogues	28	patients, clinicians	11
Xiaomeng Z 2018 (41)	China	Benefit-risk	axial spondyloarthritis	aconite preparation and tripterygium wilfordii preparation	12	--	--
Xiaomeng Z	China	Benefit-risk	rheumatoid	chuan or cao wu used alone and	13	--	--

2018 (42)			arthritis	in combination with western medicine			
Yu, Y. 2018 (43)	China	Other MCDAs	children with asthma	budesonide formulations, beclometasone formulations, fluticasone formulation	32	Pediatricians, pediatric pharmacists, pediatricians, clinical pharmacists, pharmacists, pharmaceutical specialists, pharmacoepidemiology specialists, pharmacologists, drug policy and administration specialists	25
Kolasa, K. 2018 (44)	Poland	Other MCDAs	rare diseases	--	13	representatives of the Ministry of Health, the National Health Fund, regulatory body, and patients' ombudsman	10
Weernink, M. G. M. 2018 (45)	Spain, Germany, France, Italy, UK	Other MCDAs	non-valvular atrial fibrillation	vitamin K antagonists, direct oral anticoagulants	9	patients	45
Gilabert-Perramon, A. 2017 (46)	Spain	EVIDEM	rare diseases	--	13	decision makers, evaluators, clinicians, patient representatives	16
Wagner, M. 2017 (47)	Spain Italy France	EVIDEM	radio-iodine refractory differentiated thyroid cancer	lenvatinib, watchful waiting, sorafenib	19	policy decisionmakers, specialists, patient representatives, methodologists with decision-making expertise	24

Ying L 2017 (48)	China	Benefit-risk	acute upper respiratory infection	shuanghuanglian injection, western medicine or conventional treatment	6	--	--
Angelis, A. 2017 (49)	UK	AVF	--	--	28	clinicians, nurses, pharmacologists, health economists, HTA experts, statisticians, patient representatives, policy-makers/regulators	28
Kwon, S. H. 2017 (50)	Korean	Other MCDAs	cancer	new drug A, new drug B, an existing drug	8	Korean public participants, physicians	330
Tervonen, T. 2017 (51)	UK	Other MCDAs	nonvalvular atrial fibrillation	apixaban, dabigatran, edoxaban, rivaroxaban, vitamin K antagonist	13	patients	--
Marsh, K. 2017 (52)	USA	Other MCDAs	chronic obstructive pulmonary disease	aclidinium, tiotropium	14	clinicians	6
Schey, C. 2017 (53)	Netherlands	Other MCDAs	pulmonary arterial hypertension, mucopolysaccharidosis II/VI, paroxysmal nocturnal haemoglobinuria, lennox-Gastaut	orphan drugs	9	--	--

			syndrome, myelodysplastic syndromes				
Wagner, M. 2016 (54)	Canada	EVIDEM	rare diseases	--	21	--	--
Nixon, R. 2016 (55)	Switzerland	Benefit-risk	relapsing remitting multiple sclerosis	natalizumab, placebo	11	--	--
Goetghebeur, M. M. 2016 (56)	Canada	Benefit-risk	moderate to severe plaque psoriasis	efalizumab, placebo	6	regulators, clinicians, methodologists	6
De Greef-Van Der Sandt, I. 2016 (57)	Netherlands	Benefit-risk	overactive bladder	solifenacin, mirabegron, theircombination	8	urologists, patient	--
Byun, J. H. 2016 (58)	Korean	Benefit-risk	--	placebo, cerivastatin, atorvastatin, simvastatin	4	patients	203
Yali G 2016 (59)	China	Benefit-risk	anaphylactic rhinitis	fexofenadine	19	--	--
Mohamadi, E. 2016 (60)	Iran	Other MCDAs	orthopedics	uninsured orthopedics services	9	managers, decision makers, policy makers, experts and key informants at research centers, academic centers and related organizations.	20
Al-Badriyeh, D. 2016 (61)	Qatar	Other MCDAs	gastroesophageal reflux disease, the peptic ulcers	esomeprazole, lansoprazole, pantoprazole, rabeprazole	38	gastroenterologists, internists, nephrologists, cardiologists	79
Iskrov, G. 2016	Bulgaria	Other	rare diseases	orphan drug	12	medical professionals, patient	307

(62)		MCDAs				representatives, health authorities, industry representatives	
Tervonen, T. 2015 (63)	Netherlands	Benefit-risk	cardiovascular disease	atorvastatin, fluvastatin, lovastatin, pravastatin, rosuvastatin, simvastatin	4	--	--
Hsu, J. C. 2015 (64)	Taiwan	Benefit-risk	erectile dysfunction	sildenafil, tadalafil, vardenafil	7	--	--
Hsu, J. C. 2015 (65)	Taiwan	Benefit-risk	nonvalvular atrial fibrillation	dabigatran, rivaroxaban, apizaban, warfarin	4	--	--
Migliore, A. 2015 (66)	--	Other MCDAs	osteoporosis	alendronate, risedronate, ibandronate, zolendronate, clodronate, teriparatide, denosumab, strontium ranelate	15	--	--
Tromp, N. 2015 (67)	Indonesia	Other MCDAs	HIV/AIDS	--	32	policy makers, people living with HIV/AIDS, health care workers, general population	155
Sussex, J. 2013 (68)	UK	Other MCDAs	rare diseases	--	8	patient representatives, their families and carers, payers, clinical experts, health economists	--
Ramli, A. 2013 (69)	Malaysia	Other MCDAs	hypercholesterolemia	atorvastatin, lovastatin, pravastatin, rosuvastatin, simvastatin, fluvastatin	12	drug evaluators and pharmacists, drug reviewers, hospital pharmacist, pharmacy administrator, family medicines specialist	7

Dianyou Z 2013 (70)	China	Benefit-risk	obesity	lorcaserin, placebo	12	--	--
Dianyou Z 2013 (71)	China	Benefit-risk	anaphylactic rhinitis	fexofenadine, loratadine and cetirizine	12	--	--
Goetghebeur, M. M. 2012 (72)	Canada	EVIDEM	cardiovascular, endocrinology, infectious disease, neurology, ophthalmology, oncology	10 medicines	15	health policy decision makers, clinical specialists, general practitioner, nurses, clinical pharmacists, health economists/epidemiologists	13
Hummel, M. J. M. 2012 (73)	Netherlands	Other MCDAs	depression	duloxetine, venlafaxine, selective serotonin reuptake inhibitors	11	Patients, psychiatrists, psychotherapists	16
Erjaee, A. 2012 (74)	Hong Kong	Other MCDAs	helicobacter pylori infection in children	omeprazole+amoxicillin +metronidazole+bismuth, omeprazole+amoxicillin +clarithromycin, omeprazole+amoxicillin-clavulanic acid+metronidazole	4	Physician, parents	
Youngkong, S. 2012 (75)	Thailand	Other MCDAs	chronic hepatitis B, chronic hepatitis C, severe lupus nephritis, severe asthma, urinary and fecal incontinence among disabled	treatment for chronic hepatitis B/C/severe lupus nephritis, smoking cessation program, anti-IgE, implant dentures, absorbent products for urinary and fecal incontinence, system for screening, treatment, and rehabilitation of alcoholism,	--	health professionals, academics, patients, civil society	--

			and elderly people, alcoholism, leukemia	screening for risk factors for leukemia			
Tony, M. 2011 (76)	Canada	EVIDEM	chronic non-cancer pain	tramadol	20	drug advisory committee	9
Goetghebeur, M. M. 2010 (77)	Canada	EVIDEM	turner syndrome	growth hormone	21	clinicians, ethicist, nurse, patient representatives, health economists	9
Chung, S. 2010 (78)	Korean	MAUT	--	dihydropyridine calcium channel blockers, angiotensin II receptor blockers	11	pharmacists	4
Suehs, B. T. 2009 (79)	USA, Canada	MAUT	bipolar disorder	anticonvulsant mood stabilizers, atypical antipsychotics, olanzapine-fluoxetine combination, lithium carbonate	5	psychiatric pharmacist specialists	116
Bettinger, T. L. 2007 (80)	USA	MAUT	schizophrenia	risperidone, olanzapine, quetiapine, ziprasidone, aripiprazole	4	--	--
Pérez Encinas, M. 1998 (81)	--	MAUT	intermittent claudication	pentoxifylline, buflomedil, naftidrofuryl, ticlopidine	4	--	--
Schumacher, G. E. 1991 (82)	USA	MAUT	stable angina	diltiazem, nicardipine, nifedipine, verapamil	8	--	--

Notes: "—" indicates that this item contains no content.

References

1. Zozaya N, Caballero T, González-Quevedo T, Setien PG, González MÁ, Jódar R, et al. A multicriteria decision analysis (MCDA) applied to three long-term prophylactic treatments for hereditary angioedema in Spain. Glob Reg Health Technol Assess. (2022) 9:14-21. doi: 10.33393/grhta.2022.2333.

2. Zozaya N, Abdalla F, Alfonso Zamora S, Balea Filgueiras J, Carrascosa Carrillo JM, Delgado Sánchez O, et al. Assessing the value contribution of bimekizumab for the treatment of moderate-to-severe psoriasis using a multidisciplinary reflective multi-criteria decision analysis. Expert Rev Pharmacoecon Outcomes Res. (2022) 22:941-953. doi: 10.1080/14737167.2022.2063842.

3. Gil-Nagel A, Falip M, Sánchez-Carpintero R, Abad-Sazatornil MR, Poveda JL, Aibar JÁ, et al. The contribution of fenfluramine to the treatment of Dravet syndrome in Spain through Multi-Criteria Decision Analysis. Epilepsy Behav. (2022) 132:108711. doi: 10.1016/j.yebeh.2022.108711.

4. Gasol M, Paco N, Guarga L, Bosch JÀ, Pontes C, Obach M. Early Access to Medicines: Use of Multicriteria Decision Analysis (MCDA) as a Decision Tool in Catalonia (Spain). J Clin Med. (2022) 11:1353. doi: 10.3390/jcm11051353.

5. Casellas Caro M, Hidalgo MJC, García-Erce JA, Baquero Úbeda JL, Torras Boatella MG, Gredilla Díaz E, et al. Applying reflective multicriteria decision analysis to understand the value of therapeutic alternatives in the management of gestational and peripartum anaemia in Spain. BMC Pregnancy Childbirth. (2022) 22:157. doi: 10.1186/s12884-022-04481-w.

6. Yuhan D, Huixin J, Chaojun X, Guoxun P. Evaluating vonoprazan and ilaprazole based on EVIDEM multi-criteria evidence-based decision-making framework. Chin J Hosp Pharm. (2022) 42:940-944. doi:10.13286/j.1001-5213.2022.09.13.

7. Chuqiao Y, Junzhu D, Qianqian H, Tao W, Wei L, Miao J, et al. Benefit-risk assessment of entecavir combined with three traditional Chinese medicines in treatment of chronic hepatitis B liver fibrosis. Chinese Traditional and Herbal Drugs. (2022) 53:2449-2459. doi:10.7501/j.issn.0253-2670.2022.08.022.

8. Vandewalle B, Amorim M, Ramos D, Azevedo S, Alves I, Francisco T, et al. Value-based decision-making for orphan drugs with multiple criteria decision analysis: burosumab for the treatment of X-linked hypophosphatemia. Curr Med Res Opin. (2021) 37:1021-1030. doi: 10.1080/03007995.2021.1904861.

9. Karrer L, Zhang S, Kühlein T, Kolominsky-Rabas PL. Exploring physicians and patients' perspectives for current interventions on thyroid nodules using a MCDA method. Cost Eff Resour Alloc. (2021) 19:26. doi: 10.1186/s12962-021-00279-3.

10. Bao Y, Gao B, Meng M, Ge B, Yang Y, Ding C, et al. Impact on decision making framework for medicine purchasing in Chinese public hospital decision-making: determining the value of five dipeptidyl peptidase 4 (DPP-4) inhibitors. BMC Health Serv Res. (2021) 21:807. doi: 10.1186/s12913-021-06827-0.

11. Nutt DJ, Phillips LD, Barnes MP, Brander B, Curran HV, Fayaz A, et al. A Multicriteria Decision Analysis Comparing Pharmacotherapy for Chronic Neuropathic Pain, Including Cannabinoids and Cannabis-Based Medical Products. Cannabis Cannabinoid Res. (2022) 7:482-500. doi: 10.1089/can.2020.0129.

12. Milsom I, Wagg A, Oelke M, Chapple C. Which drugs are best for overactive bladder? From patients' expectations to physicians' decisions. Int J Clin Pract. (2021) 75: e13870. doi: 10.1111/ijcp.13870.

13. Kremer IEH, Jongen PJ, Evers SMAA, Hoogervorst ELJ, Verhagen WIM, Hiligsmann M. Patient decision aid based on multi-criteria decision analysis for disease-modifying drugs for multiple sclerosis: prototype development. BMC Med Inform Decis Mak. (2021) 21:123. doi: 10.1186/s12911-021-01479-w.
14. Athanasakis K, Igoumenidis M, Boubouchairopoulou N, Vitsou E, Kyriopoulos J. Two Sides of the Same Coin? A Dual Multiple Criteria Decision Analysis of Novel Treatments Against Rheumatoid Arthritis in Physicians and Patients. Clin Ther. (2021) 43:1547-1557. doi: 10.1016/j.clinthera.2021.07.005.
15. de Andrés-Nogales F, Cruz E, Calleja MÁ, Delgado O, Gorgas MQ, Espín J, et al. A multi-stakeholder multicriteria decision analysis for the reimbursement of orphan drugs (FinMHU-MCDA study). Orphanet J Rare Dis. (2021) 16:186. doi: 10.1186/s13023-021-01809-1.
16. Angelis A, Phillips LD. Advancing structured decision-making in drug regulation at the FDA and EMA. Br J Clin Pharmacol. (2021) 87:395-405. doi: 10.1111/bcp.14425.
17. Qianqian H, Tao W, Qi K, Yang C, Liping Q, Wenjun Z. Benefit-risk assessment of traditional Chinese medicine compound preparation of Strychni Semen for rheumatoid arthritis. Chinese Traditional and Herbal Drugs. (2021) 52:495-506. doi: 10.7501/j.issn.0253-2670.2021.02.023.
18. Hao J, Zhiqi Z, Bing Z, Xiaomeng Z, Kexin G. Benefit-Risk Assessment of Mylabris Preparations in the Treatment of Primary Hepatic Carcinoma. Chinese Journal of Pharmacovigilance. (2021) 18:525-531. doi: 10.19803/j.1672-8629.2021.06.07.
19. Camps C, Badia X, García-Campelo R, García-Foncillas J, López R, Massuti B, et al. Development of a Multicriteria Decision Analysis Framework for Evaluating and Positioning Oncologic Treatments in Clinical Practice. JCO Oncol Pract. (2020) 16: e298-e305. doi: 10.1200/JOP.19.00487.
20. Sidi Y, Harel O. Comprehensive Benefit-Risk Assessment of Noninferior Treatments Using Multicriteria Decision Analysis. Value Health. (2020) 23:1622-1629. doi: 10.1016/j.jval.2020.09.002.
21. Schug S, Pogatzki-Zahn E, Phillips LD, Essex MN, Xia F, Reader AJ, et al. Multi-Criteria Decision Analysis to Develop an Efficacy-Safety Profile of Parenteral Analgesics Used in the Treatment of Postoperative Pain. J Pain Res. (2020) 13:1969-1977. doi: 10.2147/JPR.S255921.
22. de Andrés-Nogales F, Casado MÁ, Trillo JL, Ruiz-Moreno JM, Martínez-Sesmero JM, Peralta G, et al. A Multiple Stakeholder Multicriteria Decision Analysis in Diabetic Macular Edema Management: The MULTIDEX-EMD Study. Pharmacoecon Open. (2020) 4:615-624. doi: 10.1007/s41669-020-00201-2.
23. Babashov V, Ben Amor S, Reinhardt G. Framework for Drug Formulary Decision Using Multiple-Criteria Decision Analysis. Med Decis Making. (2020) 40:438-447. doi: 10.1177/0272989X20915241.
24. Angelis A, Linch M, Montibeller G, Molina-Lopez T, Zawada A, Orzel K, et al. Multiple Criteria Decision Analysis for HTA across four EU Member States: Piloting the Advance Value Framework. Soc Sci Med. (2020) 246:112595. doi: 10.1016/j.socscimed.2019.112595.
25. Angelis A, Thursz M, Ratziu V, O'Brien A, Serfaty L, Canbay A, et al. Early Health Technology Assessment during Nonalcoholic Steatohepatitis Drug Development: A Two-Round, Cross-Country, Multicriteria Decision Analysis. Med Decis Making. (2020) 40:830-845. doi: 10.1177/0272989X20940672.
26. Lin W, Qing L. Evaluation of the Benefit-risk of Different Doses of Vortioxetine in the Treatment

of Major Depression Using a Multi-criteria Decision Analysis Model. Chin J Pharmacoepidemiol. (2020) 29:77-83. doi: 10.19960/j.cnki.issn1005-0698.2020.02.001.
27. Hao J, Xiaomeng Z, Bing Z, Dan Z, Jintao L. Benefit and risk of Tripterygium Glycosides Tablets in treatment of rheumatoid arthritis based on multi-criteria decision-making analysis. China Journal of Chinese Materia Medica. (2020) 45:798-808. doi: 10.19540/j.cnki.cjcmm.20191115.502.
28. Wudong G, Xue L, Jinyu C, Yaoling W, Kun Z. A case of multi-dimensional value judgment and evaluation toolkit of pharmaceuticals. Chinese Health Resources. (2020) 23:348-351+372. doi: 10.13688/j.cnki.chr.2020.20356.
29. Guarga L, Badia X, Obach M, Fontanet M, Prat A, Vallano A, et al. Implementing reflective multicriteria decision analysis (MCDA) to assess orphan drugs value in the Catalan Health Service (CatSalut). Orphanet J Rare Dis. (2019) 14:157. doi: 10.1186/s13023-019-1121-6.
30. Guarga L, Badia X, Obach M, Fontanet M, Prat A, Vallano A, et al. Implementing reflective multicriteria decision analysis (MCDA) to assess orphan drugs value in the Catalan Health Service (CatSalut). Orphanet J Rare Dis. (2019) 14:157. doi: 10.1186/s13023-019-1121-6.
31. Baran-Kooiker, Atikeler A, Gaitova K, Holownia-Voloskova K, Turcu-Stiolica M, Kooiker A, Piniazhko C, Czech O, Marcin. Applicability of the EVIDEM Multi-Criteria Decision Analysis framework for orphan drugs- results from a study in 7 Eurasian countries. Acta Poloniae Pharmaceutica: Durg Research. (2019) 76:1.
32. Badia X, Aguarón A, Fernández A, Gimón A, Nafria B, Gaspar B, et al. Patient involvement in reflective multicriteria decision analysis to assist decision making in oncology. Int J Technol Assess Health Care. (2019) 35:56-63. doi: 10.1017/S0266462318003641.
33. Vermersch P, Martinelli V, Pfleger C, Rieckmann P, Alonso-Magdalena L, Galazka A, et al. Benefit-risk Assessment of Cladribine Using Multi-criteria Decision Analysis (MCDA) for Patients with Relapsing-remitting Multiple Sclerosis. Clin Ther. (2019) 41:249-260.e18. doi: 10.1016/j.clinthera.2018.12.015.
34. Hsu JC, Lin JY, Lin PC, Lee YC. Comprehensive value assessment of drugs using a multi-criteria decision analysis: An example of targeted therapies for metastatic colorectal cancer treatment. PLoS One. (2019) 14: e0225938. doi: 10.1371/journal.pone.0225938.
35. Mendoza-Sanchez J, Silva F, Rangel L, Jaramillo L, Mendoza L, Garzon J, et al. Benefit, risk and cost of new oral anticoagulants and warfarin in atrial fibrillation; A multicriteria decision analysis. PLoS One. (2018) 13: e0196361. doi: 10.1371/journal.pone.0196361.
36. Zozaya N, Martínez-Galdeano L, Alcalá B, Armario-Hita JC, Carmona C, Carrascosa JM, et al. Determining the Value of Two Biologic Drugs for Chronic Inflammatory Skin Diseases: Results of a Multi-Criteria Decision Analysis. BioDrugs. (2018) 32:281-291. doi: 10.1007/s40259-018-0284-3.
37. Jiménez A, Ais A, Beaudet A, Gil A. Determining the value contribution of selexipag for the treatment of pulmonary arterial hypertension (PAH) in Spain using reflective multi-criteria decision analysis (MCDA). Orphanet J Rare Dis. (2018) 13:220. doi: 10.1186/s13023-018-0966-4.
38. Roldán ÚB, Badia X, Marcos-Rodríguez JA, de la Cruz-Merino L, Gómez-González J, Melcón-de Dios A, et al. Multi-criteria decision analysis as a decision-support tool for drug evaluation: a pilot study in a pharmacy and therapeutics committee setting. Int J Technol Assess Health Care. (2018) 34:519-526. doi: 10.1017/S0266462318000569.
39. Wagner M, Samaha D, Cuervo J, Patel H, Martinez M, O'Neil WM, et al. Applying Reflective Multicriteria Decision Analysis (MCDA) to Patient-Clinician Shared Decision-Making on the

Management of Gastroenteropancreatic Neuroendocrine Tumors (GEP-NET) in the Spanish Context. Adv Ther. (2018) 35:1215-1231. doi: 10.1007/s12325-018-0745-6.
40. Wagner M, Samaha D, Khoury H, O'Neil WM, Lavoie L, Bennetts L, et al. Development of a Framework Based on Reflective MCDA to Support Patient-Clinician Shared Decision-Making: The Case of the Management of Gastroenteropancreatic Neuroendocrine Tumors (GEP-NET) in the United States. Adv Ther. (2018) 35:81-99. doi: 10.1007/s12325-017-0653-1.
41. Xiaomeng Z, Zhijian L, Bing Z, Min L. Comparison of Aconitum with Tripterygium in Treatment of Axial Spondyloarthritis: A Benefit- risk Assessment. Chinese Journal of Pharmacovigilance. (2018) 15:103-109. doi: 1672-8629 (2018) 02-0103-07.
42. Xiaomeng Z, Yongnan J, Bing Z, Ning L. Aconitum in treatment of rheumatoid arthritis: benefit-risk assessment. China Journal of Chinese Materia Medica. (2018) 43:234-241. doi: 10.19540/j.cnki.cjcmm.20171106.007.
43. Yu Y, Jia L, Meng Y, Hu L, Liu Y, Nie X, et al. Method Development for Clinical Comprehensive Evaluation of Pediatric Drugs Based on Multi-Criteria Decision Analysis: Application to Inhaled Corticosteroids for Children with Asthma. Paediatr Drugs. (2018) 20:195-204. doi: 10.1007/s40272-017-0278-5.
44. Kolasa K, Zwolinski KM, Zah V, Kaló Z, Lewandowski T. Revealed preferences towards the appraisal of orphan drugs in Poland - multi criteria decision analysis. Orphanet J Rare Dis. (2018) 13:67. doi: 10.1186/s13023-018-0803-9.
45. Weernink MGM, Vaanholt MCW, Groothuis-Oudshoorn CGM, von Birgelen C, IJzerman MJ, van Til JA. Patients' Priorities for Oral Anticoagulation Therapy in Non-valvular Atrial Fibrillation: a Multi-criteria Decision Analysis. Am J Cardiovasc Drugs. (2018) 18:493-502. doi: 10.1007/s40256-018-0293-0.
46. Gilabert-Perramon A, Torrent-Farnell J, Catalan A, Prat A, Fontanet M, Puig-Peiró R, et al. Drug evaluation and decisionmaking in catalonia: development and validation of a methodological framework based on multi-criteria decision analysis (MCDA) for orphan drugs. Int J Technol Assess Health Care. (2017) 33:111-120. doi: 10.1017/S0266462317000149.
47. Wagner M, Khoury H, Bennetts L, Berto P, Ehreth J, Badia X, et al. Appraising the holistic value of Lenvatinib for radio-iodine refractory differentiated thyroid cancer: A multi-country study applying pragmatic MCDA. BMC Cancer. (2017) 17:272. doi: 10.1186/s12885-017-3258-9.
48. Ying L, Siyue W, Chang L, Wenwen W, Yu Z, Wentao Z. Construction of Risk-benefit Assessment System for TCM Injection Based on MCDA Model: Taking Research on Shuanghuanglian Injection in Treatment of Acute Upper Respiratory Tract Infection as a Case. Chin J Pharmacoepidemiol. (2017) 26:499-510. doi:1005-0698(2017)07-0499-04.
49. Angelis A, Kanavos P. Multiple Criteria Decision Analysis (MCDA) for evaluating new medicines in Health Technology Assessment and beyond: The Advance Value Framework. Soc Sci Med. (2017) 188:137-156. doi: 10.1016/j.socscimed.2017.06.024.
50. Kwon SH, Park SK, Byun JH, Lee EK. Eliciting societal preferences of reimbursement decision criteria for anticancer drugs in South Korea. Expert Rev Pharmacoecon Outcomes Res. (2017) 17:411-9. doi: 10.1080/14737167.2017.1277144.
51. Tervonen T, Ustyugova A, Sri Bhashyam S, Lip GYH, Verdecchia P, Kwan R, et al. Comparison of Oral Anticoagulants for Stroke Prevention in Nonvalvular Atrial Fibrillation: A Multicriteria Decision Analysis. Value Health. (2017) 20:1394-1402. doi: 10.1016/j.jval.2017.06.006.
52. Marsh K, Zaiser E, Orfanos P, Salverda S, Wilcox T, Sun S, et al. Evaluation of COPD

Treatments: A Multicriteria Decision Analysis of Aclidinium and Tiotropium in the United States. Value Health. (2017) 20:132-140. doi: 10.1016/j.jval.2016.08.724.
53. Schey C, Krabbe PF, Postma MJ, Connolly MP. Multi-criteria decision analysis (MCDA): testing a proposed MCDA framework for orphan drugs. Orphanet J Rare Dis. (2017) 12:10. doi: 10.1186/s13023-016-0555-3.
54. Wagner M, Khoury H, Willet J, Rindress D, Goetghebeur M. Can the EVIDEM Framework Tackle Issues Raised by Evaluating Treatments for Rare Diseases: Analysis of Issues and Policies, and Context-Specific Adaptation. Pharmacoeconomics. (2016) 34:285-301. doi: 10.1007/s40273-015-0340-5.
55. Nixon R, Dierig C, Mt-Isa S, Stöckert I, Tong T, Kuhls S, et al. A case study using the PrOACT-URL and BRAT frameworks for structured benefit risk assessment. Biom J. (2016) 58:8-27. doi: 10.1002/bimj.201300248.
56. Goetghebeur MM, Wagner M, Nikodem M, Zyla A, Micaleff A, Amzal B. Pragmatic Multicriteria Decision Analysis (MCDA) Combined with Advanced Pharmacoepidemiology for Benefit-Risk Assessments of Medicines Adapted to the Real-Life Constraints of Regulators: Development and Case Study. Ther Innov Regul Sci. (2016) 50:620-631. doi: 10.1177/2168479016642812.
57. de Greef-van der Sandt I, Newgreen D, Schaddelee M, Dorrepaal C, Martina R, Ridder A, et al. A quantitative benefit-risk assessment approach to improve decision making in drug development: Application of a multicriteria decision analysis model in the development of combination therapy for overactive bladder. Clin Pharmacol Ther. (2016) 99:442-51. doi: 10.1002/cpt.271.
58. Byun JH, Kwon SH, Ha JH, Lee EK. A benefit-risk assessment model for statins using multicriteria decision analysis based on a discrete choice experiment in Korean patients. Ther Clin Risk Manag. (2016) 12:965-74. doi: 10.2147/TCRM.S100438.
59. Yali G, Shuqiu Z, Qing L. Application effect of multi-criteria decision model on benefit and risk assessment of fexofenadine in the treatment of allergic rhinitis. China Modern Medicine. (2016) 23:12-15. doi:1674-4721(2016)06(a)-0012-04.
60. Mohamadi E, Tabatabaei S M, Olyaeemanesh A, Sagha SF, Mobinizadeh M. Coverage Decision-Making for Orthopedics Interventions in the Health Transformation Program in Iran: A Multiple Criteria Decision Analysis (MCDA). Shiraz E Medical Journal. (2016) Inpress (Inpress). doi:10.17795/semj40920.
61. Al-Badriyeh D, Alabbadi I, Fahey M, Al-Khal A, Zaidan M. Multi-indication Pharmacotherapeutic Multicriteria Decision Analytic Model for the Comparative Formulary Inclusion of Proton Pump Inhibitors in Qatar. Clin Ther. (2016) 38:1158-73. doi: 10.1016/j.clinthera.2016.03.004.
62. Iskrov G, Miteva-Katrandzhieva T, Stefanov R. Multi-Criteria Decision Analysis for Assessment and Appraisal of Orphan Drugs. Front Public Health. (2016) 4:214. doi: 10.3389/fpubh.2016.00214.
63. Tervonen T, Naci H, van Valkenhoef G, Ades AE, Angelis A, Hillege HL, et al. Applying Multiple Criteria Decision Analysis to Comparative Benefit-Risk Assessment: Choosing among Statins in Primary Prevention. Med Decis Making. (2015) 35:859-71. doi: 10.1177/0272989X15587005.
64. Hsu JC, Tang DH, Lu CY. Risk-benefit assessment of oral phosphodiesterase type 5 inhibitors for treatment of erectile dysfunction: a multiple criteria decision analysis. Int J Clin Pract. (2015) 69:436-43. doi: 10.1111/ijcp.12548.

65. Hsu JC, Hsieh CY, Yang YH, Lu CY. Net clinical benefit of oral anticoagulants: a multiple criteria decision analysis. PLoS One. (2015) 10: e0124806. doi: 10.1371/journal.pone.0124806.

66. Migliore A, Integlia D, Bizzi E, Piaggio T. Is it the time to rethink clinical decision-making strategies? From a single clinical outcome evaluation to a Clinical Multi-criteria Decision Assessment (CMDA). Med Hypotheses. (2015) 85:433-40. doi: 10.1016/j.mehy.2015.06.024.

67. Tromp N, Prawiranegara R, Siregar A, Sunjaya D, Baltussen R. Importance of multiple criteria for priority setting of HIV/AIDS interventions. Int J Technol Assess Health Care. (2015) 31:390-8. doi: 10.1017/S0266462316000039.

68. Sussex J, Rollet P, Garau M, Schmitt C, Kent A, Hutchings A. A pilot study of multicriteria decision analysis for valuing orphan medicines. Value Health. (2013) 16:1163-9. doi: 10.1016/j.jval.2013.10.002.

69. Ramli A, Aljunid SM, Sulong S, Md Yusof FA. National Drug Formulary review of statin therapeutic group using the multiattribute scoring tool. Ther Clin Risk Manag. (2013) 9:491-504. doi: 10.2147/TCRM.S52078.

70. Dianyou Z, Yijie M, Yan L, Qing L. Evaluating the Benefit-Risk of Lorcaserin. Medical Innovation of China. (2013) 10:127-9. doi:10.3969/j.issn.1674-4985.2013.15.067

71. Dianyou Z. The Study of the Optimized Multi-Criteria Decision Analysis Model for Medicines Benefit-Risk Evaluation. Master's thesis. Shanxi Medical University. 2013.

72. Goetghebeur MM, Wagner M, Khoury H, Levitt RJ, Erickson LJ, Rindress D. Bridging health technology assessment (HTA) and efficient health care decision making with multicriteria decision analysis (MCDA): applying the EVIDEM framework to medicines appraisal. Med Decis Making. (2012) 32:376-88. doi: 10.1177/0272989X11416870.

73. Hummel MJ, Volz F, van Manen JG, Danner M, Dintsios CM, Ijzerman MJ, et al. Using the analytic hierarchy process to elicit patient preferences: prioritizing multiple outcome measures of antidepressant drug treatment. Patient. (2012) 5:225-37. doi: 10.1007/BF03262495.

74. Erjaee A, Bagherpour M, Razeghi S, et al. A Multi-Criteria Decision Making Model for Treatment of Helicobacter pylori Infection in Children. Hong Kong Journal of Paediatrics. (2012) 17:237-242. doi:10.1089/bfm.2012.9981.

75. Youngkong S, Baltussen R, Tantivess S, Mohara A, Teerawattananon Y. Multicriteria decision analysis for including health interventions in the universal health coverage benefit package in Thailand. Value Health. (2012) 15:961-70. doi: 10.1016/j.jval.2012.06.006.

76. Tony M, Wagner M, Khoury H, Rindress D, Papastavros T, Oh P, et al. Bridging health technology assessment (HTA) with multicriteria decision analyses (MCDA): field testing of the EVIDEM framework for coverage decisions by a public payer in Canada. BMC Health Serv Res. (2011) 11:329. doi: 10.1186/1472-6963-11-329.

77. Goetghebeur MM, Wagner M, Khoury H, Rindress D, Grégoire JP, Deal C. Combining multicriteria decision analysis, ethics and health technology assessment: applying the EVIDEM decision-making framework to growth hormone for Turner syndrome patients. Cost Eff Resour Alloc. (2010) 8:4. doi: 10.1186/1478-7547-8-4.

78. Chung S, Kim S, Kim J, Sohn K. Use of multiattribute utility theory for formulary management in a health system. Am J Health Syst Pharm. (2010) 67:128-35. doi: 10.2146/ajhp080672.

79. Suehs BT, Bettinger TL. A multiattribute decision model for bipolar disorder: identification of preferred mood-stabilizing medications. Am J Manag Care. (2009) 15: e42-52. doi: 10.1186/1748-5908-4-36.

80. Bettinger TL, Shuler G, Jones DR, Wilson JP. Schizophrenia: multi-attribute utility theory approach to selection of atypical antipsychotics. Ann Pharmacother. (2007) 41:201-7. doi: 10.1345/aph.1G607.
81. Pérez Encinas M, Fernández MA, Martín ML, Calvo MV, Gómez-Alonso A, Dominguez-Gil A, et al. Multicriteria decision analysis for determining drug therapy for intermittent claudication. Methods Find Exp Clin Pharmacol. (1998) 20:425-31. doi: 10.1358/mf.1998.20.5.485704.
82. Schumacher GE. Multiattribute evaluation in formulary decision making as applied to calcium-channel blockers. Am J Hosp Pharm. (1991) 48:301-8. doi: 10.1016/0014-2999(91)90044-Q.
